# Why does specialist treatment of breast cancer improve survival? The role of surgical management

**DOI:** 10.1038/sj.bjc.6601846

**Published:** 2004-04-27

**Authors:** D Kingsmore, D Hole, C Gillis

**Affiliations:** 1 University Department of Surgery, Western Infirmary, Dumbarton Road, Glasgow G11 6NT, UK; 2 West of Scotland Cancer Surveillance Unit, Department of Public Health, University of Glasgow, 1 Lilybank Gardens, Glasgow G12 8RZ, UK

**Keywords:** breast cancer, surgery, specialist, local recurrence, survival

## Abstract

Evidence that the survival of women with breast cancer treated by specialist surgeons is better than that by nonspecialists is limited. Previous reports have not identified the cause of this survival advantage. Our aim was to determine if the survival difference was due to case-mix, adjuvant treatment or the treatment provided by specialist surgeons. The case-records and pathology reports of 2776 women were reviewed. This represented 95% of all those diagnosed with breast cancer between 1/1/1986 and 31/12/1991 in a defined geographical area. Case-mix, surgery, pathology and adjuvant therapies of the 2148 women treated with curative intent were analysed. A standard of adequate surgical management was defined and confirmed as a valid predictor by examining rates of local recurrence, independent of all other prognostic factors. Against this standard, we compared the adequacy of surgical management, local recurrence rates and the survival outcomes of specialists and nonspecialists over an 8-year follow-up period. The inter-relationship between adequacy of surgical management, locoregional recurrence and survival was examined. While the case-mix and prescription of adjuvant therapies were comparable between specialist and nonspecialist surgeons, the efficacy and outcome of local treatment differed widely. Breast cancer patients treated in specialist compared to nonspecialist units had half the risk of inadequate treatment of the breast (24 *vs* 47%, *P*<0.001), a five-fold lower risk of inadequate axillary staging (8 *vs* 40%, *P*<0.001) and nine times lower risk of inadequate definitive axillary treatment (4 *vs* 38%, *P*<0.001). Local recurrence rates were 57% lower (13 *vs* 23% at eight years, *P*<0.001) and the risk of death from breast cancer was 20% lower for women treated in specialist units, after allowing for case-mix and adjuvant therapies. Adequacy of surgical management correlated with locoregional recurrence, which in turn correlated with the risk of death. The surgical management in specialised breast units is more often adequate, local and regional recurrence rates are lower, and survival is correspondingly better. We conclude that adequate surgical management of breast cancer is fundamental to improving the outcome from breast cancer irrespective of where it is delivered.

Cancer services in the UK increasingly involve specialisation, particularly in the treatment of breast cancer. Specialisation has been shown to be associated with an improvement in survival from breast cancer on a population basis but the nature of this has remained unclear (Gillis and Hole, 1996). Reasons for this lack of evidence include incomplete data on case-mix, treatment modalities and outcomes. Other studies that have similarly reported a survival advantage to treatment in larger centres could not fully account for this (Sainsbury *et al*, 1995a).

This study compares the surgical management provided by specialist and nonspecialist surgeons, the adequacy of management in relation to a guideline relevant to the study period, and their outcomes after adjustment for case-mix. The hypothesis tested is that specialist units provide better local control and that this improves survival.

## METHODS

### Identification of cases

The records of the West of Scotland Cancer Registry were used to identify all women aged under 75 years diagnosed between 1 January 1986 and 31 December 1991 with a histologically confirmed invasive breast cancer in the same defined geographical area, population 1.2 million, used in the previous study (Gillis and Hole, 1996). This time period was chosen to ensure good case-record availability with adequate follow-up to demonstrate survival differences. In addition, the implementation of the NHS Breast Screening Programme in the study area will have altered referral patterns and management after 1991. The publication of the Kings’ Fund Consensus Statement (King's Fund Forum Consensus Statement, 1986) in 1986 provided a good time-specific standard of treatment. For the 6-year period studied, 2934 women were treated for breast cancer in nine different hospitals by 54 consultant surgeons. The medical records and pathology reports of 2776 women (95% of the total) were reviewed. Excluded from the study were 72 women who had screen-detected tumours and 556 women not primarily treated with curative surgery leaving 2148 women as the basis of this study of treatment and outcome.

### Data extraction

Permission to access the medical records was granted by all consultants who treated breast cancer in the participating hospitals at that time. A higher surgical trainee with experience of treating breast cancer collected data from the following sources; in-patient records, operation notes, pathology reports, original death certificates and the case-records of the regional centre for oncology, from which all radiotherapy and chemotherapy was given. This provided information on each step of the complete process of care including case-mix, diagnostic procedures, operative procedures, pathological details, adjuvant therapies, recurrence and death.

Histological grading was not reported in all hospitals during the time period of this study. Therefore, tumours were classified into histological prognostic groups based on type and grade where mentioned: good prognosis (ductal grade I, well differentiated and special types e.g. tubular), moderate prognosis (ductal grade II, no comment, or lobular) and poor prognosis (poorly differentiated or ductal grade III). Multivariate analysis of recurrence and survival confirmed these groupings as valid, independent of tumour size and nodal status.

The presence and severity of comorbidity was assessed by the use of the Index of Co-existent Diseases, an established standardised index of comorbidity (Greenfield *et al*, 1987). Socioeconomic status was derived from a patient's postcode and grouped according to the Carstairs Index (Carstairs and Morris, 1991). Cause of death was determined from the original death certificates. Breast cancer-specific mortality was used in analysis of survival. Data on follow-up were available until the end of 1996. Average follow-up was 8 years and thus 8-year follow-up is reported.

### Definitions of specialist units

Our aim was to determine whether the previously demonstrated variation in survival between women treated by specialists and nonspecialists was related to clinical management. Thus, we kept the original classification of specialists – those with characteristics of a multidisciplinary meeting, keeping their own records, a defined relationship with a named oncologist and pathologist, and participation in clinical trials. All surgeons had equal access to the same regional centre for oncology.

### Definitions of adequacy of treatment

The clinical management of breast cancer has progressed from the time studied. Therefore, it was necessary to apply an independent standard of adequate local and regional treatment contemporary with the study period. The Kings’ Fund Consensus Statement (King's Fund Forum Consensus Statement, 1986) was published at the beginning of the study and was chosen as a standard, against which treatment could be compared. Treatment was thus categorised as adequate or inadequate. Only treatment deemed by this standard as inadequate is considered, as we were primarily concerned with factors that could adversely affect recurrence. Changes in treatment which were previously considered as excessive, for example radiotherapy after mastectomy, may now be considered as appropriate and are therefore not reported. Where treatment varied from the Kings’ Fund Guidelines, a search was made for evidence of patient choice. Excluded from the comparison of adequacy of treatment were women who chose treatment not recommended in the guidelines and women involved in clinical trials of local treatment (99 women treated in specialist units compromising 10.9% of the total treated, and 19 in nonspecialist units, 1.5% of the total treated).

Treatment of the breast was considered inadequate if breast-conserving surgery was performed for tumours larger than 30 mm, or if resection margins were positive, or if radiotherapy was omitted. Axillary staging was considered inadequate if no procedure was performed or if less than four negative nodes were obtained on an axillary sample. Axillary treatment was considered inadequate if further treatment (surgery or radiotherapy) was omitted after an inadequate staging procedure, or a positive axillary sample.

### Statistical analysis

The validity of the data set was substantiated by demonstrating the expected distribution of patient factors (age, menopausal status) and pathological factors (size, nodal status, histological prognostic grade), and confirming the recognised impact of these pathological and treatment factors (use of radiotherapy and endocrine therapy) on local recurrence and survival.

The case-mix of specialists and nonspecialists was compared using the *χ*^2^-test of association (Table 1

**Table 1:** The case-mix of operable breast cancers treated by specialists and nonspecialists

	**Specialist units**		**Nonspecialists**		
	**Number**	**(%)**	**Number**	**(%)**	***P-*value**
*Tumour size*					
0–19 mm	362	43.4	363	32.7	
20–39 mm	356	42.7	546	49.2	
40+mm	116	13.9	201	18.1	<0.001
Not stated	*72*		*132*		
Average size (mm)	22.5		25.9		
					
*Nodal status*					
Negative	470	56.4	508	54.1	
Positive	363	43.6	431	45.9	0.339
Unknown	*73*		*303*		
					
*Oestrogen receptor status*					
Negative	307	41.6	352	46.7	
Positive	431	58.4	401	53.3	0.048
Unknown	*168*		*489*		
					
*Histological prognostic group*					
Good	180	24.7	114	22.3	
Moderate	372	51.0	153	29.9	
Poor	178	24.4	244	47.7	<0.001
Not stated	*176*		*731*		
					
*Menopausal Status*					
Premenopausal	238	26.3	326	26.2	
Postmenopausal	668	73.7	916	73.8	1.000
					
*Deprivation category*					
1, 2	268	29.6	237	19.1	
3–5	355	39.2	570	45.9	
6, 7	283	31.2	435	35.0	<0.001
					
*Comorbidity*					
Incidence of FS/IDV >0	260	28.7	386	31.1	0.253
Mean IDV	1.3		1.1		
Mean FS	1.0		1.0		
					
Mean age	56.0	±10.3	56.9	±10.9	0.067

Italics refers to ‘not stated’ or ‘unknown’ categories and are not included in the calculation of %.

). The definitions of adequacy of treatment were confirmed as being valid independent of other prognostic factors (tumour size, histological prognostic group and nodal status) by comparing the relative hazard ratio (RHR) of recurrence for inadequate compared to adequate treatment on multivariate analysis using Cox's proportional hazards model (Table 2

**Table 2:** Definitions of adequacy of breast and axillary treatment and the risk of recurrence

**Treatment of the breast**		
Adequacy	Definition	RHR recurrence^a^
Adequate	Mastectomy	1.00
	Conservation: size < 30 mm+margins clear+RT	
Inadequate	Conservation: size >30 mm/margins positive/no RT	2.98
		(2.30–3.88)
		
**Treatment of the axilla**		
Adequacy	Definition	RHR recurrence^a^
Adequate	Axillary clearance/sample of 4+ negative nodes	1.00
	Axillary radiotherapy: after sampling or no surgery	
Inadequate	Axillary sample only (<4 or positive nodes)	2.29
	No axillary procedure	(1.65–3.16)

a After adjustment for nodal status, tumour size and histological prognostic group. Both *P-*values <0.001.

). The rate and adequacy of performing breast-conserving surgery, axillary staging and definitive axillary treatment were compared between specialists and nonspecialists (Table 3

**Table 3:** Specialist and nonspecialist treatment of the axilla and breast, adequacy of treatment and relative risk of recurrence

	**Specialists**		**Nonspecialists**		
	**No.**	**%**	**No.**	**%**	***P-*value**
*Treatment of the Breast*					
Conservation Surgery	462/906	51.0	572/1242	46.1	0.026
Inadequate conservation	104	22.5	285	49.8	<0.001
Local recurrence rate^a^	7%		14%		<0.001
RHR local recurrence^b^	1.00		1.54		<0.001
			(1.10–2.20)		
					
*Staging of the axilla*					
Rate of axillary staging	843/906	93.0	984/1242	79.2	<0.001
Inadequate staging	69/906	7.6	503/1242	40.5	<0.001
					
*Treatment of the axilla*					
Inadequate treatment	40/906	4.4	468/1242	37.7	<0.001
Axillary recurrence rate^b^	6%		11%		<0.001
RHR axillary recurrence^b^	1.00		2.06		<0.001
			(1.48–2.88)		
					
*Systemic treatment*					
Premenopausal women					
Chemotherapy (CT) rate	72/238	30.3	49/326	15.0	<0.001
Endocrine therapy (ET) rate	102/238	42.9	170/326	52.1	0.033
CT or ET in prognostically poor^c^	84/101	83.2	98/127	77.2	0.320
Postmenopausal women					
Endocrine therapy rate	518/668	77.5	723/916	78.9	0.537
CT or ET in prognostically poor^c^	241/282	85.5	276/332	83.1	0.440

a Actuarial estimate at 8 years.

b After adjustment for nodal status, tumour size and histological prognostic grade.

c Tumours greater than 40 mm, node positive or poor histological prognostic group.

). The 8-year breast and axillary recurrence rates were calculated using the Kaplan–Meier estimates. The RHR of recurrence comparing specialists and nonspecialists was calculated with tumour size, nodal status and histological prognostic group as independent variables. The rate of prescription of systemic therapy (endocrine therapy or chemotherapy) was compared between specialists and nonspecialists in premenopausal and postmenopausal women separately, and particularly in those with tumours of poor prognosis (larger than 40 mm, node positive, and poor histological prognostic group). The RHR of death was calculated on univariate analysis for the following variables: patient factors (age, deprivation category), pathological factors (tumour size, nodal status, histological prognostic group) and treatment factors (use of endocrine therapy, chemotherapy, after a local recurrence) and by speciality of treating surgeon (Table 4

**Table 4:** The relative hazard ratio of death comparing specialist and nonspecialists

	**Specialists**	**Nonspecialists**	
	**(baseline)**	**(RHR, 95% CI)**	***P-*value**
Unadjusted	1.00	1.37 (1.16–1.61)	<0.001
Adjusted for case mix^a^	1.00	1.35 (1.14–1.6)	<0.001
Adjusted for case mix^a^, pathology^a^	1.00	1.28 (1.01–1.49)	0.034
Adjusted for case mix^a^, pathology^a^ Treatment^a^	1.00	1.27 (1.04–1.55)	0.026
Adjusted for case mix^a^, pathology^a^, treatment^a^+adequacy^a^	1.00	1.16 (0.97–1.39)	0.103

a Independent variables: case-mix: deprivation category, age; pathology: tumour size, nodal status, histological prognostic group; treatment: endocrine therapy, chemotherapy; adequacy: adequacy of locoregional treatment.

). To allow an appreciation of the relative influence of these various factors, a multivariate analysis of the RHR of death comparing nonspecialists to specialists was calculated with all factors except local recurrence included stepwise as independent variables. This multivariate analysis was lastly repeated including all the above plus local recurrence as independent variables. To further confirm the relationship between local recurrence and death, we calculated the correlation coefficient of the RHR of recurrence against the RHR of death for each of the separate hospitals, using the best unit as baseline.

## RESULTS

### Case-mix

The incidence of locally advanced tumours at presentation was similar in specialist (116 out of 1022=11.4%) and nonspecialist (147 out of 1389=10.6%) units (*P*=0.553). Among the 906 operable cases treated by specialists and 1242 operable cases treated by nonspecialists, there was no significant difference in mean age; menopausal status; incidence and severity of comorbidity and nodal status (Table 1). The distributions of tumour size, histological prognostic group, deprivation category and oestrogen receptor status were significantly different. Nonspecialists treated women with slightly larger tumours, who were more frequently in a lower deprivation category, less frequently oestrogen receptor positive and more frequently had a poorer prognostic tumour. However, pathological stage was less completely reported in nonspecialist units; data were more often unobtainable on tumour size (8 *vs* 24%), grade (20 *vs* 61%), oestrogen receptor status (18 *vs* 38%) and axillary nodal status (7 *vs* 21% had no staging procedure).

### Diagnosis

Preoperatively, a histological diagnosis was more often obtained by specialists (81 *vs* 33%, *P*<0.001), and consequently excisional biopsy was used more frequently by nonspecialists (18 *vs* 67%, *P*<0.001). Mammography was also performed more frequently by specialist units (71 *vs* 38%, *P*<0.001).

### Confirmation of definition of adequacy as independent predictor of recurrence

In the breast or axilla, the risk of recurrence on univariate analysis was significantly higher as size increased, for node positive compared to node negative tumours, for histological prognostic group (RHR for groups: good=1 (baseline); moderate=2.55. (1.6–4.08); poor=5.19 (3.3–8.15), *P*<0.001) and for ‘inadequate’ compared to ‘adequate’ treatment (Table 2). On multivariate analysis, the RHR of breast and axillary recurrence remained significantly higher if treatment was ‘inadequate’ after including histological prognostic group, nodal status and tumour size as independent variables (RHR=2.98 and 2.29, *P*<0.001). This confirmed the validity of the definition of adequacy of treatment as an important independent predictor of recurrence.

### Treatment of the breast

Nonspecialists performed breast-conserving surgery less frequently than specialists (Table 3). Overall, inadequate breast-conserving surgery was performed twice as frequently by nonspecialists compared to specialists (23 *vs* 50%, *P*<0.001). Nonspecialists performed conservation surgery more frequently for tumours greater than 30 mm (4 *vs* 16%, *P*<0.01), left positive margins after excision (4 *vs* 12%, *P*<0.01), and omitted radiotherapy more often (16 *vs* 31%, *P*<0.01). In addition, data were often incomplete for women treated by nonspecialists (tumour size unreported in 24%, margins not specified in 42%). Specialists performed further surgery when the margins were reported as positive more frequently than nonspecialists (17 *vs* 9%, *P*<0.01). Specialists omitted radiotherapy after conservation surgery more selectively – excluding tumours with an excellent prognosis (node negative, size <10 mm, good prognostic group), specialists omitted radiotherapy after conservation surgery in 8% compared to 21% by nonspecialists (*P*<0.001).

### Staging and treatment of the axilla

The management of the axilla varied greatly between specialists and nonspecialists (Table 3, *P*<0.001. for all results). Axillary staging was more frequently omitted by nonspecialists (7 *vs* 21%), and was inadequate in half of those in whom it was attempted (503 out of 984, 51%). Likewise, definitive treatment of the axilla with surgery or radiotherapy was more frequently inadequate (4 *vs* 38%, *P*<0.001).

### Systemic treatment

In premenopausal women, specialists prescribed endocrine therapy less frequently (43 *vs* 52%, *P*=0.033), but prescribed chemotherapy twice as frequently as nonspecialists (30 *vs* 15%, *P*=0.001, Table 3). In those women with very poor prognostic tumours, who would maximally gain from systemic treatment, there was a 6% difference in the prescription of systemic therapy (17 *vs* 23% did not receive any additional treatment). In postmenopausal women, there was no difference in the rates of prescription of systemic therapy overall (78 *vs* 79%, *P*=ns), and also in those with poor prognosis. Overall, adjuvant systemic therapy was prescribed equally by specialists and nonspecialists (692 out of 906, 76% *vs* 942 out of 1242, 76%, *P*=ns).

### Recurrence rates

The local and regional recurrence rates were compared between specialists and nonspecialists (Table 3, 8-year rates and *P*<0.001 for all results). The ipsilateral breast recurrence rates of nonspecialists were double that of specialists (7 *vs* 14%, RHR=1.54). The axillary recurrence rates for nonspecialists were double that of specialists (6 *vs* 11%, RHR=2.06). The overall first locoregional (local or axillary) recurrence rate of nonspecialists was double that of specialists (13 *vs* 23%, RHR=1.59).

### Survival

The unadjusted relative risk of death from breast cancer was calculated on univariate analysis for speciality of treating surgeon (RHR=1.37, *P*<0.001, Table 4), case-mix, pathological factors, postoperative therapies, and after a local recurrence (RHR 3.42, *P*<0.0001). The RHR of death comparing nonspecialists to specialists was then calculated by adjusting for other factors in a stepwise mutlifactorial analysis: case-mix (RHR 1.35, *P*<0.001), pathological stage (RHR=1.28, *P*<0.05), adjuvant treatment (RHR=1.27, *P*<0.05). None of these factors fully accounted for the survival differences seen. To try and explain this difference, we included the adequacy of locoregional treatment. After including adequacy of locoregional treatment as an independent variable, the relative risk of death was 1.16 (0.97–1.39), (*P*=0.1), thus implying that the cause of the better survival of women treated by specialists was related to better local control of disease.

This relationship was confirmed by correlating the RHR of locoregional recurrence against the RHR of death across the nine treating hospitals using the hospital with the best outcome as a baseline. There was a strong correlation between the risk of locoregional recurrence and death (*R*^2^=0.69). There was only a poor correlation between adequacy of treatment and caseload (*R*^2^=0.24).

## DISCUSSION

This study is the most detailed and complete analysis of treatment delivered with sufficient follow-up to allow an understanding of the reasons behind variability in survival. We have demonstrated that adequacy of surgical management is an important independent risk factor for locoregional recurrence, and that the survival of women treated by surgeons with a lower locoregional recurrence rate is correspondingly better. This was independent of case-mix, prognostic pathological factors and adjuvant therapies. In specialist units during the time period studied, there was half the risk of inadequate treatment of the breast (24 *vs* 47%), one-fifth of the risk of inadequate axillary staging (8 *vs* 40%) and one-ninth of the risk of inadequate definitive axillary treatment (4 *vs* 38%). This is reflected in recurrence rates that are halved (7 *vs* 14% after breast-conserving treatment and 6 *vs* 11% in the axilla) and a 20% lower risk of death from breast cancer.

Our primary conclusion is that adequate surgical management is of underlying fundamental importance to the improvement in survival demonstrated by specialist surgeons. Categorisation using proxy measures of specialisation, such as hospital type, location, size, association with teaching facilities, caseload and selection, deflects attention away from the process of appropriate and adequate care to the structure of care delivery. However, local and regional recurrence only partly explained the associated increased risk of mortality (a halving of the relative risk of mortality in Table 4). Thus, we believe that the adequacy and appropriateness of all treatments is the key to explaining improvements in survival outcomes.

This study examined the case-records of 95% (2780 out of 2934) of all women treated for breast cancer during this time period. The completeness of identifying cases was verified from five independent sources; the cancer registry, the NHS Breast Screening service, a dedicated breast cancer regional pathology database, personal records of individual surgeons and two hospitals which held their own separate records. All data were retrieved from original case records by a surgically trained investigator (DBK) and accuracy checked by comparison with a database derived separately from pathology reports and case-records from the regional centre for oncology. The validity of the data set was confirmed both by internal checks (distribution patterns, correlation tumour size with nodal status and histological prognostic grouping, and impact of these pathological factors and treatment factors on recurrence and survival) and external comparisons. Thus, we are confident that the data is complete, accurate, and has been correctly interpreted.

Accurate pathological data was less often complete for women treated in nonspecialist units (Table 1). This was due to several factors such as the omission to surgically stage (e.g. the axilla) and the use of excisional biopsy as the method of tissue diagnosis (e.g. thus tumour size and margin status were not reported). We do not believe that the variability in staging alters our conclusions for the following reasons: firstly, we have already shown that the improvement in survival associated with specialist care cannot be due to case-mix (Gillis and Hole, 1996). Secondly, the criteria used to define inadequate treatment rely on pathological data where this is reported, thus missing information can only underestimate the true occurrence of inadequate treatment, for example excision margins were only reported in 58% of women with conservation surgery performed by nonspecialists and of these, one-fifth were positive. Thirdly, we have confirmed that our definitions of adequacy and inadequacy are strong independent factors for recurrence independent of pathology (Table 2). Thus, we do not feel that the lack of complete pathological prognostic factors renders these results void, but may illustrate the lack of importance placed upon them by those treating the patients. This also reflects the considerable debate regarding the place of surgery in the treatment of breast cancer that was prevalent at that time period.

Evidence that poorer local treatment and thus local recurrence may have contributed to poorer survival is presented in two forms; firstly using a Cox's proportional hazards model, the relative risk of dying when treated by a nonspecialist compared to a specialist remains significantly higher after inclusion of all variables (RHR=1.27), but is reduced when adequacy of local treatment is also included as an independent variable (RHR=1.16). Secondly, there is a strong correlation between the risk of death from breast cancer and the risk of local recurrence when plotted for each treating hospital (*R*^2^=0.69). Whilst these two associations are not proof that locoregional recurrence can disseminate, when taken in association with the failure of other factors to adequately account for the variation in survival between units, they do suggest that inadequate surgical treatment results in locoregional recurrence that in turn, may disseminate. These results are consistent with recently published randomised trials of postmastectomy radiotherapy in high-risk women that demonstrated increased locoregional recurrence rates and a subsequently poorer survival in women who did not receive radiotherapy (Overgaard *et al*, 1997; Ragaz *et al*, 1997).

Variation in diagnostic and therapeutic modalities has been demonstrated in many previous studies (Greenfield *et al*, 1987; Carstairs and Morris, 1991; McCarthy and Bore, 1991; Basnett *et al*, 1992; Chouillet *et al*, 1994; Lee-Feldstein *et al*, 1994; Sainsbury *et al*, 1995a, 1995b; Overgaard *et al*, 1997; Ragaz *et al*, 1997). Difficulties encountered in these studies included indirect analysis of case-records by nonsurgical staff, small numbers, incomplete analysis, limited follow-up and no measurement of outcomes. Several other surrogate measures of specialisation used in these studies have not been confirmed in this study. For example, although the rate of breast-conservation surgery in our study was similar, nonspecialists more often performed inappropriate and inadequate surgery. Similarly, chemotherapy and endocrine therapy have been used to compare treatment. In our study, both groups used chemotherapy infrequently, whereas tamoxifen was used in three-quarters of women equally. Adjuvant therapies cannot therefore be the cause of the survival differences seen, and again are poor indicators with which to compare units. Survival differences have only been reported in two major observational studies (Gillis and Hole, 1996; Sainsbury *et al*, 1995a). Neither of these studies could determine the exact treatment delivered nor critically, the adequacy of this and the impact on locoregional recurrence and survival. Despite these limitations, many guidelines have recommended that treatment of breast cancer be centralised. This study is the first to give quantitative evidence that this recommendation is justified. However, the location of services and designation of ‘specialists’ should not deflect from the fundamental importance of adequate and appropriate treatment that results in lower locoregional recurrence rates and improved survival. Indeed, other studies have found that the implementation of protocols beneficially reduces variability in treatment (Paci *et al*, 1994; Winstanley *et al*, 1995). Since the period of time studied, there have been great changes to the organisation and provision of breast cancer treatment with the implementation of the NHS Breast Screening Programme and the subsequent Quality Assurance guidelines.

Current changes in legislature now emphasise quality of care more than ever. We have demonstrated that clinical effectiveness and quality can be measured, and the emphasis on measuring quality of care will benefit patients. With the advent of clinical governance, future assessment of practice may be based on similar methodology to that used in this study.
